# Differential Effects of a Mutation on the Normal and Promiscuous Activities of Orthologs: Implications for Natural and Directed Evolution

**DOI:** 10.1093/molbev/msu271

**Published:** 2014-09-21

**Authors:** Akhil Khanal, Sean Yu McLoughlin, Jamie P. Kershner, Shelley D. Copley

**Affiliations:** ^1^Department of Molecular, Cellular and Developmental Biology and Cooperative Institute for Research in Environmental Sciences, University of Colorado Boulder

**Keywords:** molecular evolution, epistasis, ProA, enzyme evolution

## Abstract

Neutral drift occurring over millions or billions of years results in substantial sequence divergence among enzymes that catalyze the same reaction. Although natural selection maintains the primary activity of orthologous enzymes, there is, by definition, no selective pressure to maintain physiologically irrelevant promiscuous activities. Thus, the levels and the evolvabilities of promiscuous activities may vary among orthologous enzymes. Consistent with this expectation, we have found that the levels of a promiscuous activity in nine gamma-glutamyl phosphate reductase (ProA) orthologs vary by about 50-fold. Remarkably, a single amino acid change from Glu to Ala near the active site appeared to be critical for improvement of the promiscuous activity in every ortholog. The effects of this change varied dramatically. The improvement in the promiscuous activity varied from 50- to 770-fold, and, importantly, was not correlated with the initial level of the promiscuous activity. The decrease in the original activity varied from 190- to 2,100-fold. These results suggest that evolution of a novel enzyme may be possible in some microbes, but not in others. Further, these results underscore the importance of using multiple orthologs as starting points for directed evolution of novel enzyme activities.

## Introduction

Metabolic enzymes are typically prodigious catalysts, catalyzing specific reactions by up to 26 orders of magnitude (Edwards et al. 2012). These enzymes often have secondary activities, as well, that result from binding of atypical substrates in the active site in proximity to catalytic residues, metal ions, or cofactors. Secondary activities that do not affect fitness are termed promiscuous activities. Although promiscuous activities can be much less efficient than well-evolved activities (O'Brien and Herschlag 1998, 2001; Wang et al. 2003; Taylor Ringia et al. 2004) they often enhance reaction rates by orders of magnitude relative to those of uncatalyzed reactions (O'Brien and Herschlag 1998, 2001). Thus, promiscuous activities provide a reservoir of novel catalytic activities that can be recruited to serve new functions, both in nature and in the laboratory.

Here, we address a relatively unexplored aspect of promiscuity. Orthologous enzymes that serve the same function in different organisms have diverged over millions or billions of years as a result of neutral drift and possibly selection for improved activity if the activity has limited fitness of the organism. Because promiscuous activities are not under selective pressure unless they rise to a level that affects fitness, they are likely to vary as mutations accumulate. This supposition is supported by the observation that the promiscuous *N*-acyl amino acid racemase activities of the *o*-succinylbenzoate synthases from *Escherichia coli* and *Amycolaptosis* differ by more than four orders of magnitude (Palmer et al. 1999). These enzymes have diverged to the point at which there is no recognizable pairwise identity, although both belong to the enolase superfamily. However, even minor differences in sequence can result in changes in promiscuous activities. In a set of 34 variants of P450 BM3 generated by error-prone polymerase chain reaction (PCR) that had accumulated an average of four mutations but still maintained at least 75% of the parental enzyme’s activity toward 12-p-nitrophenoxydodecanoic acid, up to 4-fold changes in promiscuous activities toward five other substrates were observed (Bloom et al. 2007). Further, the patterns of reactivity toward the five other substrates differed considerably among the variants.

We predict that the effects of sequence divergence among orthologs should go beyond affecting the levels of promiscuous activities; we expect that sequence divergence will result in equally important effects on the evolvability of promiscuous activities. Differences in even a single position near the active site can influence the magnitude and even the direction of the effects of other amino acid changes. For example, substitution of Ala156 with Thr in 56 variants of the hepatitis C virus NS3 protease decreased catalytic activity in most cases. However, in 7.1% of the variants, the mutation was beneficial (Parera and Martinez 2014). The cumulative effect of epistatic interactions on successive mutations renders some evolutionary trajectories accessible, whereas many others are not. For example, of the 120 possible trajectories for accumulation of five mutations that improve the inefficient ability of TEM *β*-lactamase to hydrolyze third-generation cephalosporins, only 19 occurred by a trajectory that proceeds via only beneficial changes. In the others, fitness was decreased by at least one mutation along the trajectory (Weinreich et al. 2006).

Differences in the levels and evolvabilities of promiscuous activities have important practical consequences. Promiscuous activities that are exceedingly weak cannot serve as the precursors of new enzymes in nature unless a promoter mutation or gene duplication allows production of more enzyme or a mutation in the coding region increases the promiscuous activity so that the activity provides a fitness advantage to the organism. Thus, a potentially useful promiscuous activity may be evolvable in some organisms, but not in others. Directed evolution of promiscuous activities in the laboratory is less restricted because enzyme activity can be decoupled from organismal fitness, enzymes can be expressed at physiologically unattainable levels, and multiple mutations can be introduced simultaneously, thus allowing jumps in sequence space that avoid less beneficial intermediates. However, even with these advantages, differences in evolvability could mean the difference between the ability to evolve a useful catalyst for a practical application and a time-consuming effort that ultimately fails.

**Fig. 1.**
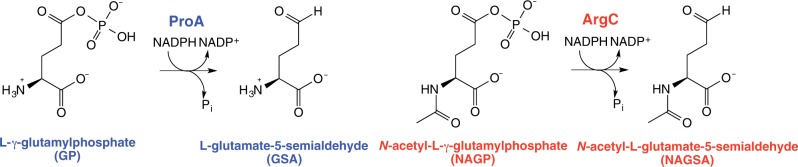
The reactions catalyzed by ProA (GP reductase) and ArgC (NAGP reductase).

Here, we begin to examine the effect of sequence divergence on the evolvability of promiscuous activities in orthologous enzymes by comparing the initial levels of a promiscuous activity in nine orthologous enzymes, and the effect of a comparable mutation on both the original and promiscuous activities of these orthologous enzymes. The model enzyme we have chosen for this investigation is L-gamma-glutamyl phosphate (GP) reductase (ProA) (see fig. 1), which is essential for synthesis of proline. ProA has a promiscuous ability to reduce *n*-acetyl-L-glutamyl phosphate (NAGP) (Yu McLoughlin and Copley 2008). NAGP reductase activity is normally supplied by ArgC, which is essential for biosynthesis of arginine during growth on glucose. ProA and ArgC are not homologous enzymes, even though both catalyze the transfer of hydride from NADPH to an acyl phosphate to form an aldehyde.

Notably, ProA cannot provide enough NAGP reductase activity to support growth of a Δ*argC* strain of *E. coli.* However, a point mutation that changes Glu383 to Ala allows ProA to serve the functions of both ProA and ArgC, even when present in a single copy in the chromosome. The change of Glu383 to Ala generates an inefficient generalist enzyme that utilizes both GP and NAGP at rates sufficient to allow Δ*argC E. coli* to grow on glucose as a sole carbon source (Yu McLoughlin and Copley 2008).

Here, we report the levels of the normal and promiscuous activities in nine orthologs of ProA. The substrate specificity of the enzymes, as measured by comparing k_cat_/K_M_ for the two substrates, varies considerably. Nevertheless, selection for an improvement in NAGP reductase activity in libraries generated by error-prone PCR resulted, in every case, in an enzyme in which the equivalent of Glu383 had been changed to Ala or Gly. The degree of improvement in the promiscuous activity achieved by the change from Glu to Ala varied from 50- to 770-fold, and was not correlated with the initial level of the promiscuous activity. Also consistent with the expectation that the effect of mutations depends upon the structural context, the decrease in the original activity caused by this amino acid change varied from 190- to 2,100-fold. Finally, enzymes for which this change decreased the original activity substantially but increased the promiscuous activity only marginally were still able to support growth of a strain lacking ArgC. This perhaps counter-intuitive result demonstrates the importance of leveling the playing field with respect to access to the active site when a single enzyme must catalyze two essential reactions.

## Results

### Kinetic Characterization of ProA Enzymes from Nine Microbes

Genes encoding ProA (GP reductase) were amplified by PCR from genomic DNA from nine microbes (*E. coli*, *Salmonella enterica* Typhimurium, *Yersinia enterocolytica*, *Allivibrio fischeri*, *Pseudomonas putida*, *Lactococcus casei*, *Bacillus subtilis*, *Methanosarcina acetivorans*, and *Saccharophagus degradans*) (see supplementary table S1, Supplementary Material online, for strain names, PCR primers and NCBI gi numbers for each enzyme). The pairwise amino acid sequence identities between these orthologs are shown in supplementary table S2, Supplementary Material online. The genes were cloned into pTrcHisB and introduced into a Δ*argC*::*kan* Δ*proA*::*cat* (DE3) strain of *E. coli* BW 25113. (This strain will be called the Δ*argC* Δ*proA* strain hereafter.) ProA enzymes were expressed in this strain to prevent contamination with *E. coli* ArgC and/or ProA during purification by nickel affinity chromatography*.* ProA undergoes slow inactivation during storage, presumably due to oxidation of the active-site cysteine, even in the presence of dithiothreitol (DTT). Thus, enzyme activities were measured immediately after purification by nickel affinity chromatography and overnight dialysis against 20 mM potassium phosphate, pH 7.5, containing 20 mM DTT.

The normal and promiscuous activities of each enzyme were assayed in the reverse direction because the substrates for the forward reactions, particularly GP, are unstable. Kinetic parameters for the L-glutamate-5-semialdehyde (GSA) dehydrogenase activities shown in table 1 have been corrected to reflect the actual concentrations of the free aldehyde substrate, which is a minor constituent of a mixture of interconverting forms. (The procedure for measuring the free aldehyde concentration is described in the supplementary material, Supplementary Material online.) Each of the enzymes has impressive specificity for its normal substrate. Table 2 shows that the ratios of k_cat_/K_M_ for the normal and promiscuous activities range from 4.1 × 10^4^ to 1.6 × 10^6^.

**Table 1.** Kinetic Parameters^a^ for GSA and NAGSA Dehydrogenase Activities of ProA Orthologs.

Source of ProA	GSA Dehydrogenase Activity			NAGSA Dehydrogenase Activity		
	k_cat_ (s^−1^)	K_M_ (mM)	k_cat_/K_M_ (M^−1^s^−1^)	k_cat_ (s^−1^)	K_M_ (mM)	k_cat_/K_M_ (M^−1^s^−1^)
*Escherichia coli*	5.1 ± 0.2	0.043 ± 0.003	1.2( ± 0.1) × 10^5^	5.2( ± 0.3) × 10^−4^	0.48 ± 0.03	1.1 ± 0.1
*Salmonella enterica*	3.7 ± 0.5	0.054 ± 0.012	6.9( ± 0.7) × 10^4^	1.1( ± 0.1) × 10^−4^	2.6 ± 0.1	0.042 ± 0.004
*Yersinia entercolitica*	7.6 ± 0.1	0.056 ± 0.002	1.34( ± 0.0) × 10^5^	1.2( ± 0.1) × 10^−4^	1.2 ± 0.1	0.095 ± 0.005
*Allivibrio fischeri*	1.9 ± 0.2	0.046 ± 0.011	4.2( ± 0.6) × 10^4^			0.048 ± 0.003
*Pseudomonas putida*	0.74 ± 0.23	0.14 ± 0.09	6.1( ± 1.7) × 10^3^	1.0( ± 0.3) × 10^−3^	5.2 ± 1.9	0.15 ± 0.055
*Lactococcus casei*	6.0 ± 1.0	0.23 ± 0.05	2.6( ± 0.1) × 10^4^	1.4( ± 0.1) × 10^−4^	0.49 ± 0.14	0.30 ± 0.058
*Bacillus subtilis*	1.7 ± 0.2	0.15 ± 0.02	1.1( ± 0.1) × 10^4^	1.4( ± 0.3) × 10^−3^	6.0 ± 1.5	0.24 ± 0.015
*Methanosarcina acetivorans*	0.43 ± 0.03	0.014 ± 0.004	2.5( ± 1.1) × 10^4^			0.025 ± 0.002
*Saccharophagus degradans*	0.40 ± 0.04	0.085 ± 0.013	4.7( ± 0.4) × 10^3^	3.0( ± 0.3) × 10^−5^	0.82 ± 0.16	0.037 ± 0.004

^a^Values given are the average of three replicates and the corresponding standard errors. Only values for k_cat_/K_M_ are given when the enzyme could not be saturated with the substrate.

**Table 2.** Ratio of Values for k_cat_/K_M_ for the GSA and NAGSA Dehydrogenase Activities of Wild Type and Mutant ProA Enzymes.

	Wild-Type ProA	ProA* (“E383A”)
*Escherichia coli*	1.1 × 10^5^	0.41
*Salmonella enterica*	1.6 × 10^6^	17.6
*Yersinia entercolitica*	1.4 × 10^6^	2.2
*Allivibrio fischeri*	8.8 × 10^5^	1.1
*Pseudomonas putida*	4.1 × 10^4^	24
*Lactococcus casei*	8.7 × 10^4^	7.4
*Bacillus subtilis*	4.6 × 10^4^	0.4
*Methanosarcina acetivorans*	1.0 × 10^6^	10.6
*Saccharophagus degradans*	1.3 × 10^5^	1.2

Values of the k_cat_/K_M_ for the GSA dehydrogenase activity of the various orthologs varied by a factor of 40. This difference is striking, although perhaps not surprising, given the different environments inhabited by these microbes and the consequent differences in growth rate, nutrient availability, and selection pressures. We considered the possibility that expression in *E. coli* might affect the catalytic parameters of the purified enzymes, perhaps due to subtle defects in protein folding caused by suboptimal codon usage. We were able to obtain a small amount of the *P. putida* ProA after expression in *P. putida.* Although the values of k_cat_ and K_M_ varied somewhat compared with those obtained for the enzyme after expression in *E. coli*, the value for k_cat_/K_M_ was not substantially different.

### Directed Evolution of the Promiscuous NAGP Reductase Activities of Orthologous ProAs

Libraries of each *proA* were generated using mutagenic PCR and cloned into pTrcHisB. Libraries containing approximately 10^5^ members were introduced into the Δ*argC* Δ*proA* strain and spread onto plates containing M9/glucose supplemented with 1 mM proline. Plasmids were recovered from individual colonies after growth on M9/glucose overnight at 37°C and the mutant *proA* genes were sequenced. Each variant had one or a few mutations. A change converting the equivalent of *E. coli* Glu383 to either Ala or Gly was found in every one of the 34 genes sequenced (see table 3). In several cases, this was the only change relative to the parent sequence.

**Table 3.** Amino Acid Changes Identified in ProA Orthologs that Supported Growth of Δ*argC* Δ*proA E. coli* on M9/Glucose When Expressed on pTrcHisB.

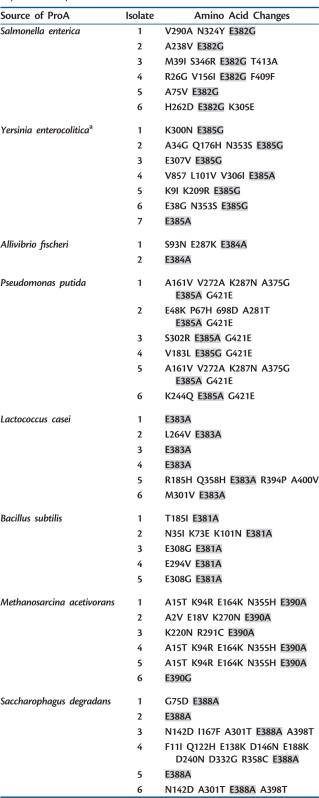

Note.—Changes in residues corresponding to Glu383 in *E. coli* are highlighted in gray.

^a^The *Y. enterocolitica* gene used as the parental sequence had a mutation relative to the published sequence that changed Ser333 to Gly. Each variant in the table maintained this change.

We generated mutant versions of eight orthologs in which only the Glu corresponding to *E. coli* Glu383 was changed to Ala. (These variants will be referred to as ProA* hereafter.) Each gene was cloned into pTrcHisB, and the resulting plasmids were introduced into the Δ*argC* Δ*proA* strain. Aliquots of the transformed cells were grown on M9/glucose agar containing ampicillin and 0.5 mM isopropyl β-D-1-thiogalactoctopyranoside (IPTG) at 37 °C. All of the enzymes were able to support growth under these conditions.

### A Change of “Glu383” to Ala Has Different Effects on the GSA and NAGSA Dehydrogenase Activities of the Mutant ProA Enzymes

**Fig. 2.**
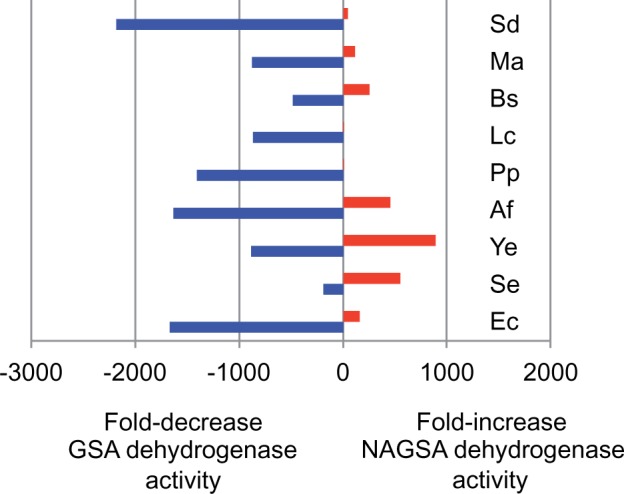
Fold-changes in k_cat_/K_M_ for the GSA dehydrogenase (blue) and NAGSA dehydrogenase (red) activities caused by the change of the equivalent of Glu383 in *Escherichia coli* ProA to Ala in ProA orthologs. Ec, *E. coli;* Se, *Salmonella enterica*; Ye, *Yersinia enterocolitica*; Af, *Allivibrio fischeri*; Pp, *Pseudomonas putida*; Lc, *Lactococcus casei*; Bs, *Bacillus subtilis*; Ma, *Methanosarcina acetivorans*; Sd, *Saccharophagus degradans*.

We assayed the GSA and *N*-acetyl-L-glutamate-5-semialdehyde (NAGSA) dehydrogenase activities of the orthologous ProA* enzymes (see table 4). The effect of the change of the equivalent of Glu383 to Ala is markedly different among the enzymes. Figure 2 shows the fold-changes in k_cat_/K_M_ caused by the mutation. (Note that although we measure k_cat_/K_M_ in the reverse direction, fold-changes in k_cat_/K_M_ in the forward direction will be equal to those in the reverse direction [Yu McLoughlin and Copley 2008].)

**Table 4.** Kinetic Parameters for GSA and NAGSA Dehydrogenase Activities for ProA Orthologs in Which the Equivalent of Glu383 Has Been Changed to Ala^a^.

Source of ProA	GSA Dehydrogenase Activity			NAGSA Dehydrogenase Activity		
	k_cat_ (s^−1^)	K_M_ (mM)	k_cat_/K_M_ (M^−1^s^−1^)	k_cat_ (s^−1^)	K_M_ (mM)	k_cat_/K_M_ (M^−1^s^−1^)
*Escherichia coli*	0.022 ± 0.004	0.32 ± 0.04	69 ± 5	0.079 ± 0.001	0.46 ± 0.03	172 ± 12
*Salmonella enterica*	0.024 ± 0.001	0.065 ± 0.012	370 ± 41	0.014 ± 0.001	0.66 ± 0.11	21 ± 3
*Yersinia entercolitica*	0.018 ± 0.001	0.11 ± 0.01	164 ± 6.2	3.7( ± 0.2) × 10^−3^	0.052 ± 0.008	73 ± 10
*Allivibrio fischeri*			26 ± 5	1.4( ± 0.1) × 10^−3^	0.065 ± 0.023	23 ± 5
*Pseudomonas putida*			4.6 ± 0.2			0.19 ± 0.017
*Lactococcus casei*			31 ± 1			4.2 ± 0.7
*Bacillus subtilis*			23 ± 2	2.8( ± 0.4) × 10^−3^	0.046 ± 0.019	61 ± 14
*Methanosarcina acetivorans*	1.8( ± 0.5) × 10^−3^	0.056 ± 0.028	32 ± 15			3.5 ± 0.9
*Saccharophagus degradans*			2.2 ± 0.2			1.9 ± 0.3

^a^Values given are the average of three replicates and the corresponding standard errors. Only values for k_cat_/K_M_ are given when the enzyme could not be saturated with the substrate.

### ProA* Enzymes Can Support Growth of the Δ*argC* Δ*proA* (DE3) Strain on Glucose Even When Expressed from a Single-Copy Gene

The experiments described above were initially designed to examine the evolvability of the NAGP reductase activity of ProA orthologs using selection for growth on M9/glucose in the presence of proline. However, each ProA* ortholog also supported growth of the Δ*argC* Δ*proA* strain on M9/glucose in the absence of proline when expressed from the multicopy plasmid pTrcHisB, even in the absence of IPTG.

We recloned the genes encoding each ProA* into the single-copy plasmid pETcoco2 to determine whether they were able to support growth of the Δ*argC* Δ*proA* strain on M9/glucose when expressed at more physiologically relevant levels. In the presence of 0.5 mM IPTG, expression of each orthologous ProA* except that from *A. fischeri* enabled growth on M9/glucose plates without proline or arginine to a diameter of 0.5 mm within 3–5 days (data not shown). Leaky expression of the *E. coli* ProA* in the absence of IPTG allowed growth to a diameter of 0.5 mM in one day. However, in the presence of 0.5 mM IPTG, growth of the strain expressing the *E. coli* ProA* was poor; colonies reached a size of 0.5 mm in eight days. Apparently overexpression of the *E. coli* ProA* is toxic. A similar effect was seen in liquid cultures; the lag phase was increased by approximately 8-fold in the presence of 0.5 mM IPTG (data not shown).

**Fig. 3.**
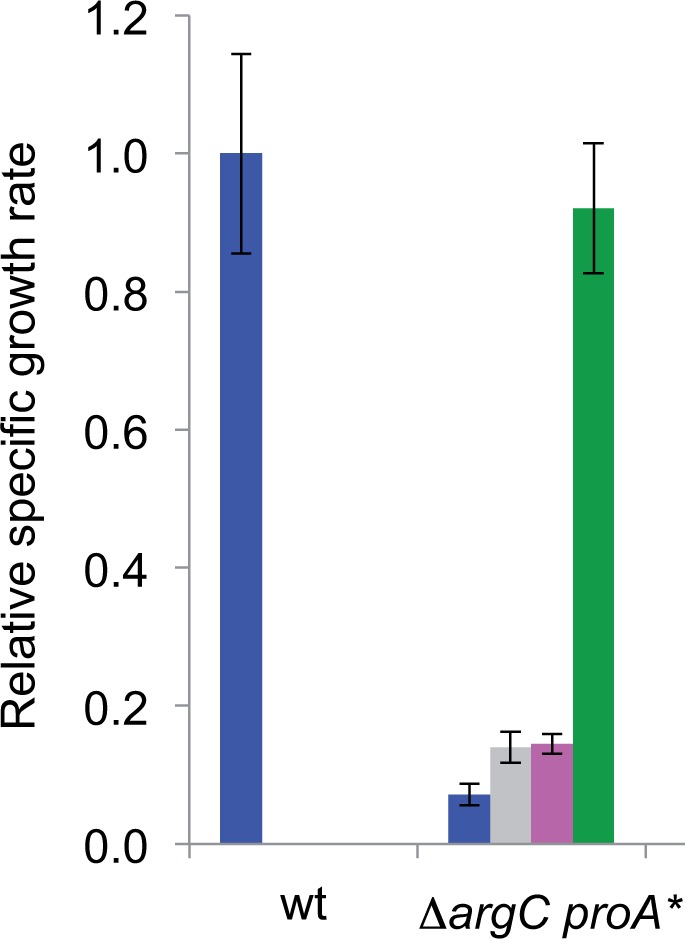
The effect of supplementation with proline and/or arginine on the growth rate of a Δ*argC* strain in which E. coli proA* has been integrated into the genome in place of *proA.* Blue, M9/glucose; gray, M9/glucose + 0.4 mM proline; magenta, M9/glucose + 5.2 mM arginine; green, M9/glucose + 0.4 mM proline and 5.2 mM arginine. Wt, wild type *E. coli* BW25113.

To investigate the performance of *E. coli* ProA* under physiological conditions, we integrated E. coli proA* into the genome in place of *proA* in the Δ*argC* strain so that expression would be under control of the native promoter and in the actual genomic context. Figure 3 shows that this strain grows on glucose at only 7% the rate of wild type *E. coli.* Addition of both proline and arginine increases growth rate to that of the wild-type strain. However, addition of either proline or arginine alone increases the growth rate only slightly.

## Discussion

The ProA enzymes that were examined in this work derive from microbes that, in some cases, diverged more than 3 billion years ago. The most closely related enzymes from *E. coli* and *S. enterica* are 86% identical. The most divergent enzyme in the set is from the Archaeon *M. acetivorans* and is no more than 45% identical to any of the other orthologs.

All of the ProA orthologs we examined had a promiscuous NAGP reductase/NAGSA dehydrogenase activity, the activity normally supplied by ArgC. This finding is not surprising given the similarity in the chemical reactions catalyzed by these two enzymes, which involve transfer of a hydride to an acyl phosphate on substrates that differ only in whether the amino group is acetylated or not. However, it is curious that ProA and ArgC are not evolutionarily related; they have no detectable pairwise sequence identity and crystal structures of the two enzymes (PDB 1O20 and 1VKN, respectively) from *Thermatoga maritima* have different folds. Apparently nature has come up with two independent architectures for catalysis of this simple reaction. We expect that there is no reason that the ProA scaffold could not serve as a starting place for evolution of an efficient NAGP reductase; there are many known examples of convergent evolution in which proteins with different scaffolds have evolved to catalyze identical reactions, sometimes using different mechanisms. Examples include tyrosine decarboxylases (Kezmarsky et al. 2005), fructose-1,6-bisphosphate aldolases (Murzin et al. 1995), and pantothenate kinases (Yang et al. 2006).

Each ProA shows an impressive level of specificity for its normal substrate (see table 2), preferring the normal substrate over the promiscuous substrate by more than four orders of magnitude. This specificity has likely evolved to prevent binding of NAGP to the active site so that it does not interfere with reduction of GP.

**Fig. 4.**
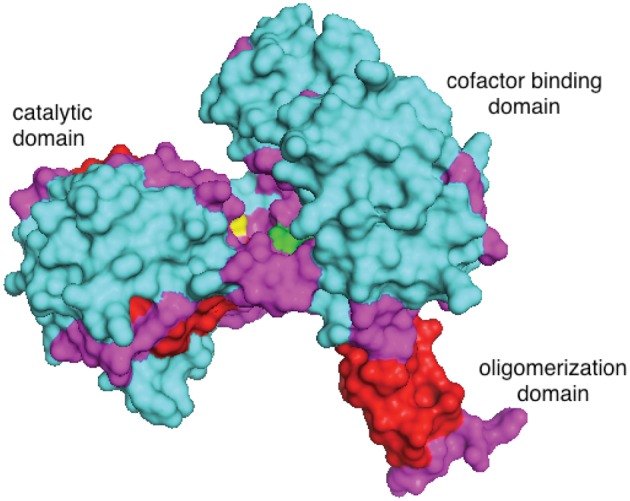
The structure of ProA from *T. maritima* (PDB 1O20) showing Glu381 in green and the catalytic Cys255 in yellow. Cyan, alpha helices; red, beta sheets; magenta, loops.

Given the sequence divergence among our set of orthologous enzymes, we were particularly interested in whether different mutations would be required to increase the NAGP reductase activity of orthologous ProAs. To our surprise, the equivalent of Glu383 in the *E. coli* enzyme was changed to either Ala or Gly in every case. This mutation is clearly critical in adapting the active site to accommodate NAGP. Glu381 in the *T. maritima* enzyme (PDB 1O20) (the equivalent of *E. coli* Glu383) is within 9 Å of the catalytic cysteine (see fig. 4). Although there are no liganded structures of ProA that reveal the actual position of the substrate in the active site, it is tempting to speculate that replacement of a negatively charged residue in the active site of ProA with a smaller neutral residue generates additional room in the active site for the acetyl group on the amino group of NAGP. We previously found that this change did not affect the affinity of NAGSA to the active site in the absence of ATP (Yu McLoughlin and Copley 2008). However, many kinases undergo conformational changes that close the active site cleft when both substrates are bound (Schulz et al. 1990; Larsen et al. 1997; Nishimasu et al. 2007). Thus, the smaller residue at position 383 may allow proper positioning of NAGSA in the closed form of the enzyme.

We initiated our directed evolution experiments using a selection for an increased ability to provide arginine in a Δ*argC* Δ*proA* strain. We cloned libraries generated by error-prone PCR of each ortholog into pTrcHisB, introduced the libraries into the Δ*argC* Δ*proA* strain, and selected for growth on glucose supplemented with proline. We discovered that the change of the equivalent of Glu383 to Ala in each ortholog allowed growth of the Δ*argC* Δ*proA* strain on glucose supplemented with proline. Further investigation showed that this change also allowed growth of the Δ*argC* Δ*proA* strain on glucose without supplemental proline. When the level of expression was decreased to a more physiological level by expression from the single-copy vector pETcoco2 in the presence of 0.5 mM IPTG, seven of the eight ProA* orthologs were still able to support growth of the Δ*argC* Δ*proA* strain on glucose. Although expression of these seven orthologs led to different rates of growth on glucose in liquid medium, we did not attempt to correlate the in vitro properties of the enzymes with growth rate for two reasons. First, the codon usage in the various *proA* genes was not necessarily optimal for *E. coli*, and the levels of expression of the enzymes varied considerably (data not shown). More importantly, the GP substrate is channeled from the active site of glutamyl kinase (ProB) to the active site of ProA (Smith et al. 1984). We expect that the orthologous ProA* enzymes will vary in their ability to interact with *E. coli* ProB, and that this variation will affect the partitioning of the enzyme’s in vivo activities between production of GSA and NAGSA.

Integration of E. coli proA* into the genome of the Δ*argC* strain under control of the native promoter yields a strain that can grow on glucose, but grows quite slowly due to limitations in its ability to synthesize both arginine and proline (see fig. 3). However, the Δ*argC* strain itself cannot grow on glucose at all. Although the change of Glu383 to Ala significantly compromises the normal activity of the enzyme (see fig. 2), the trade-off between the GP reductase and NAGP reductase activities is clearly necessary to restore viability when arginine cannot be obtained in any other way.

**Fig. 5.**
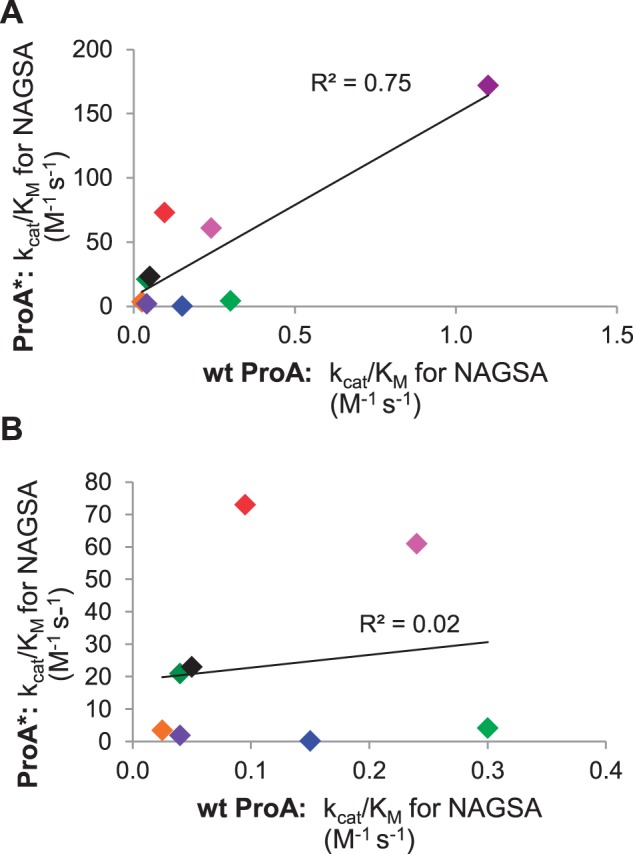
The improvement in k_cat_/K_M_ for NAGSA achieved by the change in the equivalent of *Escherichia coli* Glu383 to Ala does not correlate well with the k_cat_/K_M_ for NAGSA of the wild type enzyme. (*A*) Regression using data points for all nine orthologs; (*B*) regression using all data points except that for *Escherichia coli.* (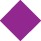) *E. coli* E383A ProA; (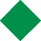) *Salmonella enterica* E382A ProA; (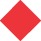) *Yersinia enterocolitica* E385A ProA; (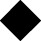) *Allivibrio fischeri* E384A ProA; (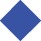) *Pseudomonas putida* E385A ProA; (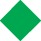) *Lactococcus casei* E383A ProA; (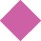) *Bacillus subtilis* E381A ProA; (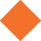) *Methanosarcina acetivorans* E390A ProA; (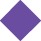) *Saccharophagus degradans* E388A ProA.

Although the equivalent mutation appears to have been required for every ortholog to improve synthesis of arginine in vivo, both the level of improvement of the promiscuous activity and the degree of impairment of the original activity due to the single mutation varied widely (see fig. 2). This finding is in agreement with the expectation that the effect of a comparable mutation will vary depending upon the structural context. It is notable that the effect of changing the equivalent of Glu383 to Ala differs substantially even between the most closely related enzymes, those from *E. coli* and *S. enterica.* Figure 5 shows a plot of the NAGSA dehydrogenase activity of each ProA* as a function of the same activity in the wt ProA. When all of the data are considered, there appears to be a correlation between the NAGSA dehydrogenase activities of each orthologous ProA and ProA*. However, the point for the *E. coli* enzymes is clearly an “influential point” whose value dominates the regression. When the point for *E. coli* is removed, there is no longer any correlation between the NAGSA dehydrogenase activities of the ProA and ProA* in the remaining orthologs.

The results reported here demonstrate principles that are important for both evolution of new enzymes in nature and directed evolution of new enzymes in the laboratory. Directed evolution of promiscuous activities is one of the core technologies that underpins the field of synthetic biology. Synthetic biologists can take advantage of promiscuous activities as the starting point for directed evolution of novel catalysts for use in designed pathways for synthesis of pharmaceuticals and chemicals or degradation of toxic pollutants. These novel enzymes enable green technologies that can supplant environmentally harmful processes that rely on petrochemical feedstocks, organic solvents and toxic heavy metal catalysts. Novel enzymes can also be used to expand the pharmaceutical repertoire by allowing stereospecific and stereoselective transformations of natural or synthetic products. A better understanding of the evolvability of promiscuous activities will enhance the success of all of these important applications.

Although we analyzed only the first step in an evolutionary trajectory, it is clear that the effects of comparable mutations varies among orthologs, and the enzyme with the highest initial activity may not be the best choice for directed evolution studies. Further, other properties, particularly stability, that are important for evolvability (Bloom et al. 2006; Tokuriki et al. 2008) may also vary among orthologs. The results reported here suggest that efforts to evolve new enzyme activities in the laboratory should be initiated with more than one ortholog, because the enzyme that has the highest level of a promiscuous activity may not be as evolvable as an ortholog that has a rather poor level of the same activity.

The findings reported here also have important implications with respect to evolution of new functions in nature, particularly when the only enzyme that can provide a promiscuous activity that is important for survival in a challenging new environment is one that already serves an essential function. Our demonstration that a mutation that severely compromises the original activity occurs in multiple orthologous enzymes under selection for a secondary activity underscores the importance of a trade-off in catalytic efficiency when a single enzyme is needed to provide two essential reactions. Although decreasing the efficiency of the normal reaction hinders production of an essential metabolite, it also increases the ability of the secondary substrate to access the active site. In this regard, the kinetic parameters of the *P. putida* ProA* are particularly interesting; the change of Glu385 to Ala has a negligible effect on the promiscuous activity, but decreases the normal activity by 1,400-fold. Thus, elevation of the flux through a promiscuous reaction to a physiologically significant level can be accomplished simply by diminishing competition from the normal substrate.

When a single enzyme is responsible for catalyzing two essential reactions, the fitness of the organism is not related to the levels of each activity in a simple way. The flux through each reaction will depend upon the concentration of the enzyme, the relative values of k_cat_/K_M_ for the two reactions, and the concentrations of the substrates for each reaction. In this case, in which an inefficient enzyme is required for synthesis of both proline and arginine, fitness will be a function of the levels of those amino acids in the medium as well as the levels of flux through each reaction that are achievable in the cytoplasm.

## Materials and Methods

### Bacterial Strains and Culture

Electrocompetent 10*β* cells and XL1-Blue Supercompetent cells were obtained from New England Biolabs and Agilent, respectively. Deletion of *proA* from *E. coli* BW25113 Δ*argC*::*kan* (strain JW3930_1 from the Keio collection) was described previously (Yu McLoughlin and Copley 2008). *Escherichia coli* BW25113 (*lacI*^q^ *rrnB*_T14_ Δ*lacZ*_WJ16_ *hsdR514* Δ*araBAD*_AH33_ Δ*rhaBAD*_LD78_) is a derivative of the F^-^, λ^-^, *E. coli* K-12 strain BD792 (Wanner 1983). A DE3 cassette encoding T7 RNA polymerase under control of the *lacUV5* promoter was introduced into the BW25113 Δ*argC*::*kan* strain using a Novagen λ (DE3) lysogenization kit. The DE3 cassette, which encodes T7 RNA polymerase and LacIq, enables expression of genes under control of T7 and related weakened promoters (such as the trc and lac UV 5 promoters [Tegel et al. 2011]) upon addition of IPTG. A strain in which *proA* in *E. coli* BW25113 Δ*argC*::*kan* was replaced by proA* was constructed using gene gorging (Herring et al. 2003). 

Starter cultures of the Δ*argC* Δ*proA* strain of *E. coli* BW 25113 were grown in LB medium (Cold Spring Harbor Protocols 2006, available at: http://cshprotocols.cshlp.org/site/recipes, last accessed October 10, 2014) (5 ml) containing ampicillin (100 µg/ml). After overnight growth at 37 °C, the cultures were subjected to centrifugation at 3,600 × g for 20 min at 4 °C. The cell pellet was washed five times with 1 ml of H_2_O and then used to inoculate 150 µl of M9 medium (Cold Spring Harbor Protocols 2010, available at: http://cshprotocols.cshlp.org/site/recipes, last accessed October 10, 2014)) containing glucose (20 mM) and ampicillin (50 µg/ml) when pTrcHisB or pETcoco-2 was present in the cells. The cells were grown with shaking at 37 °C in a VarioScan plate reader (Thermo Electron Corporation) in M9 medium (2010) containing glucose (20 mM) or on agar plates made in the same medium with 50 µg/ml ampicillin.

### Cloning of *proA* Orthologs

Genes encoding ProA were amplified by PCR from genomic DNA of nine microbes using the primers listed in supplementary table S1, Supplementary Material online and cloned into pTrcHisB (Life Technologies). Each gene was again amplified by PCR from the corresponding pTrcHisB plasmid and cloned into pETcoco2 (Novogen).

### Purification of ProA

His-tagged ProA enzymes were expressed in Δ*argC* Δ*proA E. coli* cells to prevent contamination by wild type *E. coli* ProA or ArgC. Proteins were purified by nickel affinity chromatography as described in the Supplementary Material online.

### Assays for GSA and NAGSA Dehydrogenase Activities

NAGSA and GSA dehydrogenase activities were measured by monitoring the appearance of NADPH at 340 nm in reaction mixtures containing 100 mM potassium phosphate, pH 7.6, 1 mM NADP^+^, varying concentrations of NAGSA or GSA, and catalytic amounts of ProA or ProA*. All kinetic measurements were done at room temperature. True values for K_M_ cannot be obtained because NAGSA exists in equilibrium with its hydrate, and GSA is a minor component in an equilibrating mixture of GSA, its hydrate, and the intramolecular cyclization product 1-pyrroline 5-carboxylate. Apparent values of K_M_ based upon the total concentration of GSA and its hydrate were calculated based upon the experimental determination of the concentration of GSA and its hydrate in stock solutions as described in the supplementary material, Supplementary Material online.

### Directed Evolution of ProA

Mutagenic PCR employing Mutazyme II (Agilent) was performed to amplify *proA* genes from the pTrcHisB into which they had been cloned. The purified PCR fragments were digested with NheI and BamHI and religated into pTrcHisB (Life Technologies). The plasmid was then introduced into electrocompetent 10*β* cells (New England Biolabs), and the cells plated on LB agar containing 50 µg/ml ampicillin. The cells were grown overnight at 37°C. The following morning, about 10^5^ colonies were suspended in 1 ml of LB and plasmid DNA was extracted using a Qiagen plasmid mini kit.

The resulting libraries were introduced into the Δ*argC* Δ*proA* strain by electroporation. Transformants were plated on M9/glucose containing proline (1 mM) and incubated overnight at 37°C. The next day, the largest colonies were picked from each plate and plasmid DNA was isolated. The inserted *proA* genes were sequenced by Macrogen.

### Site-Directed Mutagenesis of *proA*

Wild-type *proA* alleles from each strain were changed to introduce a codon for Ala at a position corresponding to E383 in the *E. coli* enzyme using the QuikChange protocol (Agilent). The products were digested by DpnI, and the plasmids were introduced into XL1-Blue Supercompetent cells (Agilent). The cells were plated on LB agar containing 50 µg/ml ampicillin and grown overnight at 37°C. The introduction of the intended mutation was verified by sequencing purified plasmid DNA.

## Supplementary Material

Supplementary material, methods, and tables S1 and S2 are available at *Molecular Biology and Evolution* online (http://www.mbe.oxfordjournals.org/).

Supplementary Data
