# Supplementary material for: Differential Effects of a Mutation on the Normal and Promiscuous Activities of Orthologs: Implications for Natural and Directed Evolution
Source: Mol Biol Evol. 2014 Sep 21;32(1):100–8. doi: 10.1093/molbev/msu271 (PMC4271523; doi:10.1093/molbev/msu271)
Supplement: Supplementary Data [file supp_32_1_100__index.html]

Differential Effects of a Mutation on the Normal and Promiscuous Activities of Orthologs: Implications for Natural and Directed Evolution — Differential Effects of a Mutation on the Normal and Promiscuous Activities of Orthologs: Implications for Natural and Directed Evolution — Supplementary Data 

# Differential Effects of a Mutation on the Normal and Promiscuous Activities of Orthologs: Implications for Natural and Directed Evolution

## Supplementary Data

files

**Files in this Data Supplement:**

- Supplementary Data - pdf file
