# Supplementary material for: Differential Effects of a Mutation on the Normal and Promiscuous Activities of Orthologs: Implications for Natural and Directed Evolution
Source: Mol Biol Evol. 2014 Sep 21;32(1):100–8. doi: 10.1093/molbev/msu271 (PMC4271523; doi:10.1093/molbev/msu271)
Supplement: Supplementary Data [file supp_msu271_Supplementary_MaterialsfinalMBE.pdf]

Supplementary Table 1. Orthologs of *E. coli* ProA used in this study.

Organism	PCR primers	NCBI gi
Salmonella enterica subsp. *enterica* serovar Choleraesuis (ATCC 10708)	Forward: 5'GAATCGCTAGCATGCTGGAACAAATGGGCATTGCT3' Reverse: 5'AAGCTTGGATCCTTACGCACGAATCGTACCATCGCC3'	3332740
Yersinia enterocolitica (ATCC 23715)	Forward: 5'GAATTCGCTAGCATGAGCATGCTGGAACAGATGGGGAAA3' Reverse: 5'AAGCTTGGATCCTTAGCTACGAACTAAATCATCGCC3'	4715101
Aliivibrio fischeri (ATCC 700601)	Forward: 5'GAATTCGCTAGCATGGGTCAAGCAGCTAAAGCCGCT3' Reverse: 5'AAGCTTGGATCCTTAAGAACGGATTAGATAATCCGC3'	3277391
Pseudomonas putida KT2440 (ATCC 47054)	Forward: 5'GAATTCGCTAGCATGACTGAGTCCGTTCTTGACTAC3' Reverse: 5'AAGCTTGGATCCTCAGCAGGACTCCTGGCC3'	24986570
Lactococcus casei (ATCC 334)	Forward: 5'GAATTCGCTAGCATGGACGCAACGACAATTGATCTT3' Reverse: 5'AAGCTTGGATCCTCAATTTTTCCGGATCTGCCCATT3'	6406539
Bacillus subtilis (ATCC 23857)	Forward: 5'GAATTCGCTAGCATGAGTGAAGTTTCTGTAAAGCG3' Reverse: 5'AAGCTTGGATCCCTATTCACGGATTTGCCCAGTTCC3'	936166
Methanosarcina acetivorans (ATCC 35395)	Forward: 5'GAATTCGCTAGCATGGCTGAAGATATTGAAACTAAAGTTG3' Reverse: 5'AAGCTTGGATCCTTAATTCACATCCGCAAACTTAAG3'	1475994
Saccharophagus degradans (ATCC 43961)	Forward: 5'GAATTCGCTAGCATGCGCGATCTTTATGCAAAACGA3' Reverse: 5'AAGCTTGGATCCTTAGTTGCGAATGTGACCGTCGCC3'	3965894

Supplementary Table 2. Pairwise sequence identities between the orthologous ProA enzymes[a] used in this study.

	Ec	Se	Ye	Af	Pp	Lc	Bs	Ma	Sd
Ec	100	86	73	59	44	51	46	40	46
Se		100	72	57	44	51	47	39	46
Ye			100	62	44	48	46	40	44
Af				100	43	45	47	41	44
Pp					100	47	44	45	66
Lc						100	52	40	47
Bs							100	40	45
Ma								100	44
Sd									100

[a]Ec, *E. coli;* Se, *S. enterica*; Ye, *Y. enterocolitica*; Af, *A. fischeri*; Pp, *P. putida*; Lc, *L. casei*;, Bs, *B. subtilis*; Ma, *M. acetivorans*; Sd, *S. degradans*.

Supplementary Methods

Cloning of *proA* genes. *proA* genes were amplified from genomic DNA of various bacteria (obtained from ATCC except for *S. degradans*, which was available in the laboratory) using primers designed to introduce restriction sites as needed for cloning (see Supp. Table 1) and the following amplification protocol: Step 1, 95 °C for 2 min; Step 2, 95 °C for 30 s; Step 3, 50 °C for 30 s; Step 4, 72 °C for 1 min 30 s; Step 5, repeat steps 2-4 30 times; Step 6, 72 °C for 5 min. The PCR products were digested with DpnI for 1 hr at 37 °C to remove the genomic DNA. For cloning into pTrcHisB, the PCR product was digested with NheI and BamHI and gel-purified using a Qiagen QIAquick gel extraction kit. The vector was linearized by digestion with NheI and BamHI at 37 °C for 14 hrs and then gel-purified using a Qiagen gel extraction kit. The *proA* insert was ligated into the linearized vector using T4 DNA ligase (NEB) at 37 ºC for 30 minutes. This construction results in incorporation of Met-Gly_2-Ser-His_6-Gly-Met-Ala-Ser before the initial Met of ProA. For cloning into pETcoco-2, the sequences encoding the tagged ProA enzymes were amplified from the corresponding pTrcHis constructs using the following primers: forward: 5' CAG CCT GAT ACA GAT TAA ATC AGA GCG GCC GCA TCG 3'; reverse : 5' CGA TGC GGC CGC TCT GAT TTA ATC TGT ATC AGG CTG 3'. The amplified fragments were then digested with NheI and NotI for 5 hr at 37 ºC. The resulting fragments were ligated into pETcoco-2, which had been linearized by digestion with NheI and NotI, using DNA ligase for 20 min at 16 °C.

Generation of competent cells. Five mL cultures of Δ*argC::kan* Δ*proA::cat* (DE3) cells were grown overnight at 37 °C in LB containing 50 μg/mL kanamycin. The next morning, the cells

were harvested by centrifugation at 9,500 x g for 5 min at 4 ºC. The cell pellet was resuspended in 100 μL of LB. Five μL of this cell suspension was inoculated into 500 mL of LB containing 50 μg/mL kanamycin. The cells were grown at 37 °C until the OD_{600} reached 0.6. The cultures were incubated on ice for 20 min prior to centrifugation at 3800xg for 15 min at 4 °C. The cells were washed with 500 mL of 10% glycerol. The pellet was resuspended in 50 mL of 10% glycerol and centrifuged at 3800 x g for 15 min at 4 °C. The pellet was then resuspended in 5 mL of 10% glycerol and centrifuged at 1,900 x g for 15 min at 4 °C. The cell pellet was resuspended in 1 mL of 10% glycerol. Fifty μL aliquots were flash frozen in liquid nitrogen and stored at -80 °C.

Purification of ProA enzymes. pTrcHis plasmids encoding *proA* alleles were introduced into competent Δ*argC::kan* Δ*proA::cat* (DE3) cells by electroporation and the transformants were spread onto LB plates containing 100 μg/mL ampicillin. After growth, a single colony was inoculated into 5 mL of LB containing 100 μg/mL ampicillin and the cells were grown with shaking for 14 hrs at 37 °C. The cells were harvested by centrifugation at 9,500 x g for 5 min. The cell pellet was resuspended in 1 mL LB. A 100 µL aliquot of the cell suspension was inoculated into 1 L of LB containing 100 µg/mL ampicillin. IPTG was added to a concentration of 1 mM when the OD_{600} was 0.7 and the culture was grown with shaking for 14 hrs at 37 ºC. The cells were harvested by centrifugation at 3800 x *g* for 15 min at 4 °C. The cell pellet was resuspended in lysis buffer (50 mM sodium phosphate, pH 8.0, 10 mM imidazole, 300 mM sodium chloride, 20 mM DTT) containing 10% glycerol (2 mg cells per mL of buffer) and stored at -80 °C. The suspended cells were lysed by two passes through a French press at 12000 psi at 4 °C. The lysate was subjected to centrifugation for 20 min at 11,000 x *g* at 4 °C. His-tagged

enzymes were purified as described in the Ni-NTA Purification System Handbook (Invitrogen). The supernatant was loaded onto a 12 cm x 2 cm glass column containing 8-10 mL of Ni-NTA agarose (Invitrogen) that had been pre-equilibrated with lysis buffer. The column was washed with 20 mL of 50 mM sodium phosphate, pH 8.0, containing 20 mM imidazole, 300 mM sodium chloride and 20 mM DTT, and the enzyme was eluted with 50 mM sodium phosphate, pH 8.0, containing 300 mM imidazole, 300 mM sodium chloride and 20 mM DTT. Fractions containing the protein were identified by measuring the OD_{280}, pooled and dialyzed overnight at 4 ºC against 20 mM potassium phosphate, pH 7.5, containing 20 mM DTT. Proteins were concentrated by ultrafiltration using a 10,000 MWCO Microcon filtration device (Millipore). Protein purity was confirmed by SDS-PAGE. Protein concentrations were determined by the Bradford assay (Bradford 1976) using bovine serum albumin as a standard.

An improved assay for GSA and NAGSA dehydrogenase activities. The normal and promiscuous activities of ProA are assayed in the reverse direction because the physiological reactants, glutamyl phosphate (GP) and N-acetylglutamyl phosphate (NAGP), are unstable. In particular, GP cyclizes rapidly to form 5-oxo-proline.

Assays in the reverse direction are complicated by the equilibration of GSA and NAGSA with their hydrated forms, as well as the equilibration of GSA with its intermolecular cyclization product *S*-1-pyrolline-5-carboxylate (P5C). This equilibrium strongly favors P5C at neutral pH (Mezl and Knox 1976; Bearne and Wolfenden 1995). According to Bearne and Wolfenden (Bearne and Wolfenden 1995), the hydrated form of GSA represents about 4% of the total concentration of GSA+hydrate+P5C at pD 7.4, and the free aldehyde itself represents < 1% of the acyclic form.

The kinetic parameters we reported previously (Yu McLoughlin and Copley 2008) were apparent parameters based upon the total concentration of GSA and P5C. The true substrate for the enzyme should be GSA, as it is the free aldehyde that is produced from G5P and NADPH in the forward direction. For this work, we measured the concentration of GSA (in the hydrate and free aldehyde forms) in the solution in order to obtain an improved value of k_{cat}/K_M for each ProA. We added a large amount of *E. coli* ProA (15 μM) to a solution of NAGSA or GSA in 100 mM potassium phosphate, pH 7.6, containing 1 mM $NADP^+$, in 1 mL. The absorbance at 340 nm due to formation of NADPH exhibited a burst followed by a linear phase. The magnitude of the burst was proportional to the total amount of GSA and P5C, whereas the slope of the linear phase was constant, regardless of the amount of GSA and P5C. We conclude that the burst represents consumption of the free aldehyde and hydrated form of the substrate, which we assume are in rapid equilibrium, while the linear phase represents the slower rate at which the P5C ring opens to form GSA. The magnitude of the burst, which typically represented 1-2% of the total concentration of GSA+P5C, was measured before each set of kinetic assays.

NAGSA and GSA dehydrogenase activities were measured by monitoring the appearance of NADPH at 340 nm in reaction mixtures containing 100 mM potassium phosphate, pH 7.6, 1 mM $NADP^+$, varying concentrations of NAGSA or GSA, and catalytic amounts of ProA or ProA*. All kinetic measurements were done at room temperature. Apparent values of K_M based upon the total concentration of GSA+hydrate+P5C were adjusted based upon the concentration of GSA+hydrate measured as described above.

Purification of *N*-succinyldiaminopimelate aminotransferase/acetylornithine transamine (ArgD). *E. coli argD* was cloned into pET-21d in order to add a sequence encoding an N-

terminal His_6-tag and the resulting plasmid was introduced into electrocompetent *E. coli* DH5α cells (New England Biolabs) according to the manufacturers protocol. Transformants were selected on LB plates containing 100 μg/mL of ampicillin. A single colony from the plate was inoculated into 5 mL LB containing ampicillin (100 μg/mL) and the culture was grown overnight with shaking at 37 °C. Plasmid DNA was purified using the QiaPrep Spin Miniprep protocol (Qiagen). The purified plasmid was introduced into 50 μL of 10β cells (New England Biolabs). The cells were allowed to recover for 1 hr at 37 °C in 1 mL of SOC medium (New England Biolabs). A 50 μL aliquot was then spread onto a plate of LB agar containing 50 μg/mL ampicillin. After overnight growth at 37 ºC, a single colony was used to inoculate 5 mL of LB containing 100 μg/mL ampicillin. The cells were grown overnight at 37 °C with shaking. The following morning, the cells were harvested by centrifugation at 4 °C for 15 min at 1900 x g. The cell pellet was resuspended in 100 μL of LB, and a 50 μL aliquot was inoculated into 500 mL of LB containing 100 μg/mL ampicillin. The cells were grown until the OD_{600} was 0.6-0.8, at which time IPTG was added to a final concentration of 1 mM. Cell growth was continued for an additional 3 hrs. The cells were harvested by centrifugation at 3800xg for 15 min at 4 °C. ArgD was purified using the protocol described above for purification of ProA.

Synthesis of N-acetylglutamate 5-semialdehyde. NAGSA was synthesized enzymatically using N-succinyldiaminopimelate aminotransferase/acetylornithine transaminase (ArgD) in a 300 mL reaction mixture containing 20 mM potassium phosphate, pH 8.5, 100 mM *N*-acetyl ornithine, 100 mM α-ketoglutarate, 0.01 mM pyridoxal-5'-phosphate, and 50-100 mg ArgD. After incubation for 5 hrs at 37 ºC, the reaction mixture was loaded at room temperature onto a 500 mL Dowex 50 column that had been prepared by washing with 200 mL of 5M H_2SO_4 followed

by washing with H_2O until the pH of the eluate was no longer acidic. NAGSA and α-ketoglutaric acid do not bind to the resin and were collected in 50 mL fractions. Fractions containing NAGSA were identified using 2-aminobenzaldehyde following the procedure of Vogel and Davis (Vogel, H.J. and Davis, B.G. 1952 *JACS* 74, 109) and pooled. The pH was increased to 4 by the addition of KOH. The mixture was then loaded at room temperature onto a 200 mL Dowex 1 column that had been pre-equilibrated with 250 mL of 1 M NaOH and washed with 2 L of water. The column was washed with 300 mL of water and then eluted with 200 mM Na_2SO_4. Fractions containing NAGSA were pooled and the pH was lowered to 4 using H_2SO_4. The solution was concentrated by rotary evaporation until the concentration of NAGSA was between 200 – 250 mM. Sulfate was removed by adding solid $Ba(OH)_2$ until no further precipitate was formed. The precipitated $BaSO_4$ was removed via centrifugation for 10 min at 1900 x g at 4 °C. The final NAGSA solution was stored in 1 mL aliquots at -80 °C.

Synthesis of Glutamate Semialdehyde. GSA was synthesized enzymatically using ArgD in a 200 mL reaction containing 20 mM potassium phosphate, pH 8.5, 35 mM L-ornithine, 12 mM α-ketoglutaric acid, 0.01 mM pyridoxal-5'-phosphate, and 50-100 mg ArgD. After overnight incubation at 37 °C, the reaction mixture was loaded onto a 500 mL Dowex 50 column that had been pre-equilibrated with 200 mL of 5 M H_2SO_4 and subsequently washed with 2 L of water at room temperature. The column was washed successively with 500 mL of water, 200 mL of 50 mM H_2SO_4, and 300 mL of 100 mM H_2SO_4. GSA was eluted with 600 mM H_2SO_4. Fractions containing GSA were identified using 2-aminobenzaldehyde as previously described (Mezl and Knox 1976) and pooled. The solution was concentrated by rotary evaporation until the total concentration of GSA and P5C was about 9 mM. (Further concentration of GSA results in

irreversible polymerization reactions (Mezl and Knox 1976).) Sulfate was removed as described above. One mL aliquots of GSA were stored at -80 °C.

Generation of *proA* libraries. *proA* orthologs were amplified from the pTrcHisB plasmids into which they had been cloned using error-prone PCR with Mutazyme II by the following amplification protocol: Step 1, 95 °C for 2 min; Step 2, 95 °C for 30 s; Step 3, 50 °C for 30 s; Step 4, 72 °C for 1 min 30 s; Step 5, repeat steps 2-4 30 times; Step 6, 72 °C for 5 min. The PCR products were digested with NheI, BamHI and DpnI overnight at 37 °C and then purified by gel extraction prior to ligation into pTrcHisB that had been linearized by digestion with Nhe1 and BamHI. Ligation was carried out at 10 ºC overnight. The libraries were introduced into electrocompetent 10-β cells. The transformants were incubated in 1 mL SOC medium (New England Biolabs) at 37 ºC with shaking for one hour prior to plating 200 μL aliquots onto LB agar containing amplicillin (100 μg/mL). Tens of thousands of colonies from each plate were recovered in LB medium and plasmids were isolated from each sample. Each library was introduced into Δ*argC*::*kan* Δ*proA*1*cat* cells by electroporation. After the transformants were allowed to recover in LB at 37 ºC for one hour with shaking, the cells were recovered, washed twice with PBS and resuspended in 200 μL PBS. A 1 μL aliquot was spread onto agar plates containing LB and ampicillin (100 μg/mL); in each case, more than 10^4 colonies grew. The remaining cells were spread onto agar plates containing M9/glucose and 1 mM proline. Plasmids were isolated from several colonies that grew on the M9/glucose/proline plates and the inserted *proA* genes were sequenced.

Bearne SL and Wolfenden R. 1995. Glutamate gamma-semialdehyde as a natural transition state analogue inhibitor of Escherichia coli glucosamine-6-phosphate synthase. *Biochemistry* 34: 11515-11520.

Bradford M. 1976. A rapid and sensitive method for the quantitation of microgram quantities of protein utilizing the principle of protein-dye binding. *Anal Biochem* 72: 248-254.

Mezl VA and Knox WE. 1976. Properties and analysis of a stable derivative of pyrroline-5-carboxylic acid for use in metabolic studies. *Anal Biochem* 74: 430-440.

Yu McLoughlin S and Copley SD. 2008. A compromise required by gene sharing enables survival: implications for evolution of new enzyme activities. *Proc Natl Acad Sci USA* 105: 13497-13502.
